# Computational Molecular Networks and Network Pharmacology

**DOI:** 10.1155/2017/7573904

**Published:** 2017-11-08

**Authors:** Kang Ning, Xinming Zhao, Ansgar Poetsch, Wei-Hua Chen, Jialiang Yang

**Affiliations:** ^1^Key Laboratory of Molecular Biophysics of the Ministry of Education, College of Life Science and Technology, Huazhong University of Science and Technology, Wuhan, Hubei 430074, China; ^2^Institute of Science and Technology for Brain-Inspired Intelligence, Fudan University, Shanghai 200433, China; ^3^Plant Biochemistry, Ruhr-University Bochum, Bochum, Germany; ^4^School of Biomedical and Healthcare Sciences, Plymouth University, Plymouth, UK; ^5^Department of Genetics and Genomic Sciences, Icahn School of Medicine at Mount Sinai, New York City, NY 10029, USA

For biomedical research relying on systems biology approaches, two major subdomains might have profound impacts: (1) the molecular network for understanding the principles of regulations at multiple levels and (2) network pharmacology for investigating the effect of small molecules on gene dynamics. In this issue, we lay emphasis on analytical method development for molecular networks and network pharmacology for a broad area of applications. Any computational methods towards better interpretation of molecular networks, as well as those methods related to network pharmacology, would be desired. This special issue has been affiliated with the workshop “Molecular Networks and Network Pharmacology” on the IEEE International Conference on Bioinformatics and Biomedicine (BIBM), which was held on December 15–18, 2016, in Shenzhen, Guangdong, China.

In this special issue, we have received 28 papers, out of which 17 have been accepted for publication. These papers could be categorized into four types: (1) computational approaches for biological network analysis, (2) statistical approaches for biological network analysis, (3) network pharmacology studies focusing on cancer therapy, and (4) network pharmacology studies focusing on Traditional Chinese Medicine.

We would like to lay emphasis on three works which are of special interest for this special issue. Firstly, the work by K. Huang et al. from Ohio State University on single-cell gene expression patterns, which has proposed the virtual analytical approach for single-cell sorting analysis, has advanced our knowledge on single-cell categorization as well as heterogeneities among single cells. Secondly, in the work done by J. Gao et al. from Beijing University of Chemical Technology, deep learning approach (Convolutional Neural Networks (CNN)) has been applied on calling structural variations based on low coverage data. Thirdly, the work by Y. Ga et al. from Tibetan Traditional Medical College on network pharmacology of a Traditional Chinese Medicine (i.e., TCM, which is Wuwei-Ganlu-Yaoyu-Keli) has shown us a nice example of how network pharmacology could be applied for new principles of TCM.

As our editorial team all can agree, both areas of computational molecular networks and network pharmacology, and more importantly their interplay, have represented a rapidly growing interdisciplinary research field. In this field, both computational and experimental approaches have been used for network modeling, based on which better understanding and applications of existing drugs (western drugs and TCM) could be explored. With the advancement of deep learning and the urgent need for drug development, we believe that the topics included in this special issue would be of great interest for those working in related areas, as well as for general audience. Thus, we hope that the readers will enjoy this special issue.

*Kang Ning* *Xinming Zhao* *Ansgar Poetsch* *Wei-Hua Chen* *Jialiang Yang*

